# Non-coding RNA and pseudogenes in neurodegenerative diseases: “The (un)Usual Suspects”

**DOI:** 10.3389/fgene.2012.00231

**Published:** 2012-10-31

**Authors:** Valerio Costa, Roberta Esposito, Marianna Aprile, Alfredo Ciccodicola

**Affiliations:** Institute of Genetics and Biophysics ‘Adriano Buzzati-Traverso’, National Research CouncilNaples, Italy

**Keywords:** miRNA, pseudogenes, ceRNA, neurodegeneration, cancer

## Abstract

Neurodegenerative disorders and cancer are severe diseases threatening human health. The glaring differences between neurons and cancer cells mask the processes involved in their pathogenesis. Defects in cell cycle, DNA repair, and cell differentiation can determine unlimited proliferation in cancer, or conversely, compromise neuronal plasticity, leading to cell death and neurodegeneration. Alteration in regulatory networks affecting gene expression contribute to human diseases onset, including neurodegenerative disorders, and deregulation of non-coding RNAs – particularly microRNAs (miRNAs) – is supposed to have a significant impact. Recently, competitive endogenous RNAs (ceRNAs) – acting as sponges – have been identified in cancer, indicating a new and intricate regulatory network. Given that neurodegenerative disorders and cancer share altered genes and pathways, and considering the emerging role of miRNAs in neurogenesis, we hypothesize ceRNAs may be implicated in neurodegenerative diseases. Here we propose, and computationally predict, such regulatory mechanism may be shared between the diseases. It is predictable that similar regulation occurs in other complex diseases, and further investigation is needed.

## INTRODUCTION

Neurodegenerative diseases (NDs) are assuming a growing relevance in the pathological scenario that jeopardizes human health. Since degenerative processes are closely age-related, NDs incidence is stalking the increment of life expectancy in all industrialized countries. These common and complex disorders are mainly characterized by the selective and progressive death of one or more specific neuronal populations, and an elevated number of cases is represented by Alzheimer’s, Parkinson’s, and Huntington’s diseases (AD, PD, and HD, respectively). Although the increasing interest in exploring neurodegenerative phenomena and mechanisms has led to significant progresses, deciphering the molecular basis of NDs is far from complete. The identification of causative mutations in very rare monogenic Mendelian forms of NDs has provided only clues to interpret their pathological basis. Most of NDs forms rely on the combination of multiple genetic and environmental factors, and the onset and severity are influenced by their complex interactions (Ertekin-Taner, 2011). Thus, exclusively investigating risk factors and mutations in genes responsible of NDs monogenic forms may be reductive.

Regulatory multilayer networks affecting gene expression are emerging as relevant contributors in the etiology of human diseases, including NDs. Particularly, a growing number of studies are showing deregulation of different classes of non-coding RNAs (ncRNAs) – microRNAs (miRNAs), long intergenic (lincRNAs) and long non-coding RNAs (lncRNAs) – suggesting they may have a relevant impact on disease onset/progression (Esteller, 2011). Their involvement in a variety of biological processes related to neurogenesis and neurodegeneration – such as synaptic plasticity – has been demonstrated (Qureshi and Mehler, 2012).

Recently, and rather unexpectedly, NDs are displaying similarities at different levels with cancer. Epidemiological studies suggest an association between NDs’ incidence and a reduced (or increased) risk of specific cancers, although conflicting results have been reported (Plun-Favreau et al., 2010). Cancer cells go through uncontrolled divisions and show unlimited proliferative potential, whereas neurons degeneration implies progressive loss of synaptic structure or function and substantial cell death. At first glance it might seem paradoxical that a plethora of molecules may be common to both diseases, even though dramatic changes in transcriptional and post-transcriptional regulation similarly occur in both cancer cells and degenerating neurons. Accordingly, miRNAs have been reported in both conditions as key regulators, exerting their inhibiting roles either on common genes involved in cancer and neurodegeneration, either on different genes belonging to common pathways. Moreover, the same pool of miRNAs can target distinct genes involved in pathways specific of each disease (Du and Pertsemlidis, 2011).

Therefore, since carcinogenesis-related processes and neuronal circuits functionality involve not only common molecules, but also multiple similar regulatory mechanisms, here we hypothesize these complex disorders may also share a recently described mechanism of gene expression regulation based on miRNAs unbalance. Indeed, it has been recently demonstrated in cancer that some transcribed pseudogenes share miRNA responsive elements (MREs) with their parent genes competing for the same miRNAs (Poliseno et al., 2010; Karreth et al., 2011; Tay et al., 2011). LncRNAs have the same ability of acting as miRNAs sponges (Cesana et al., 2011; Salmena et al., 2011). Since each miRNA is predicted to regulate up to hundreds of targets, altered expression of such transcripts – named competitive endogenous RNAs (ceRNAs) – may disrupt the equilibrium of available miRNAs, in turn modifying mRNAs abundance. Given such considerations, we speculate that ceRNA mechanism, demonstrated in cancer, may also be involved in NDs etiology.

Therefore, to evaluate the potential impact of ceRNA-mediated regulation of gene expression in NDs, we independently analyzed pseudogenes and their parent genes, with evidence of differential expression in AD, PD, and HD, disclosing predicted miRNAs binding sites common to pseudogene/gene pairs. Similarly, we identified a restricted pool of miRNAs targeting lncRNAs differentially expressed in such diseases. Our analysis suggests these deregulated non-coding transcripts (both pseudogenes and lncRNAs) may act as ceRNAs. Thus, we propose a new regulatory mechanism – common to neurodegenerative and cancer processes – may exist, and it cannot be excluded that a similar regulatory network may also underlie other human complex diseases.

## miRNA FUNCTION

MicroRNAs, endogenously expressed small (20–25 nucleotides) single-stranded RNAs, play crucial roles in the post-transcriptional regulation, binding a short region (seed) of mRNAs – a complementary sequence usually located in 3′ UTRs – and consequently leading to transcripts’ degradation (Guo et al., 2010) or repressing their translation (Bartel, 2009). Since each miRNA can target thousand of genes and, *vice versa*, each gene can be targeted by several miRNAs (Rajewsky and Socci, 2004; Rajewsky, 2006), such molecules are crucially implied in the fine-tuned regulation of gene expression. The proven involvement of miRNAs both in physiological and pathological processes has rapidly exposed them to the spotlight, shifting the research focus toward this class of ncRNAs^1^ (Packer et al., 2008; Patel et al., 2008; Martí et al., 2010; Margis et al., 2011; Miñones-Moyano et al., 2011; Chan and Kocerha, 2012; Geekiyanage et al., 2012).

### miRNA IN NEURODEGENERATION

Understanding brain functionality has always represented a fascinating and challenging goal. Nonetheless, its complex structure and inaccessibility have made extremely difficult to study neurodegenerative processes. The identification of causative mutations explains only a little percentage of ND cases (Sutherland et al., 2011), whereas the alteration of gene expression levels and epigenetic changes, are emerging as new contributors to neurodegenerative disorders. Indeed, AD and PD can be seen as “gene-dosage effect” disorders: AD could be caused by gene duplication of Aβ precursor protein (*APP*; Podlisny et al., 1987; Rovelet-Lecrux et al., 2006), likewise α-synuclein *locus* duplication or triplication causes PD (Singleton et al., 2003; Chartier-Harlin et al., 2004). Thus, it is reasonable to speculate that altered levels of some crucial transcripts may have a dramatic impact on neurons functionality.

Specific patterns of miRNAs expression in restricted areas have been documented in brain development and senescence (Miska et al., 2004; Kapsimali et al., 2007). In the past few years, a growing number of reports have shown that precursor and mature miRNA transcripts and miRNA processing machinery itself (Drosha and Dicer) are disrupted during ND progression (Hébert et al., 2009; Ghose et al., 2011; Schofield et al., 2011). In particular, gene expression analyses of sporadic PD (Kim et al., 2007) and AD (Lukiw, 2007; Cogswell et al., 2008) revealed that miRNA deregulation is associated to neurodegeneration, and that some miRNAs repress *APP* expression (Long and Lahiri, 2011; Liu et al., 2012), although discordant results suggest that some experimental and technical concerns still exist (discussed in Costa et al., 2010, 2012).

Nonetheless, the hypothesis that miRNAs are involved in ND etiology is intriguing, and understanding how, and at what extent, they contribute to neurodegenerative processes remains a crucial endpoint.

## ceRNA THEORY

Competition among different classes of RNAs for a pool of miRNAs has been first suggested, then demonstrated, by both theoretical and experimental studies (Seitz, 2009; Poliseno et al., 2010; Karreth et al., 2011; Tay et al., 2011). Seitz (2009) proposed that many computationally identified miRNA target genes might represent some “non-legitimate targets,” or low-affinity miRNAs “pseudotargets.” Therefore, such mRNAs would act as competitive inhibitors of miRNAs, by preventing their binding to legitimate targets.

In the wake of such hypothesis, the “competing endogenous RNAs” theory (Salmena et al., 2011) has proposed the existence of legitimate *bona fide* miRNA competitors, such as demonstrated for the gene/pseudogene pairs* PTEN*/*PTENP1* and *KRAS/KRAS1P* (Karreth et al., 2011; Tay et al., 2011). mRNAs can talk each other through their 3′ UTRs, and the “indirect interactions” can regulate their expression levels. Such transcribed – but untranslated – regions contain MREs which can regulate *in cis* the transcript levels itself and *in trans* can alter the levels of different pools of miRNAs, consequently affecting the levels of other mRNAs. Such theory, experimentally confirmed in a mouse model of melanoma (Karreth et al., 2011; Tay et al., 2011), proposes that virtually all types of RNA can communicate each other through a new fascinating “biological alphabet,” in which MREs are the “letters” whose different combinations may form an entire universe of “words” (Licatalosi et al., 2008; Chi et al., 2009).

### PSEUDOGENES IN NEURODEGENERATIVE DISEASES

The contribution of ceRNAs to the availability of miRNAs in the cell has been established in cancer, and their altered expression modifies the abundance of mRNAs (Poliseno et al., 2010; Tay et al., 2011). Thus, understanding the contribution of ceRNAs on gene expression deregulation is particularly relevant not only in different tumors but also in other human complex diseases. In particular, since recent evidences show NDs share common altered genes, pathological mechanisms, and cellular processes with cancer, we decided to address whether ceRNAs may contribute also to NDs pathogenesis.

**FIGURE 1:**
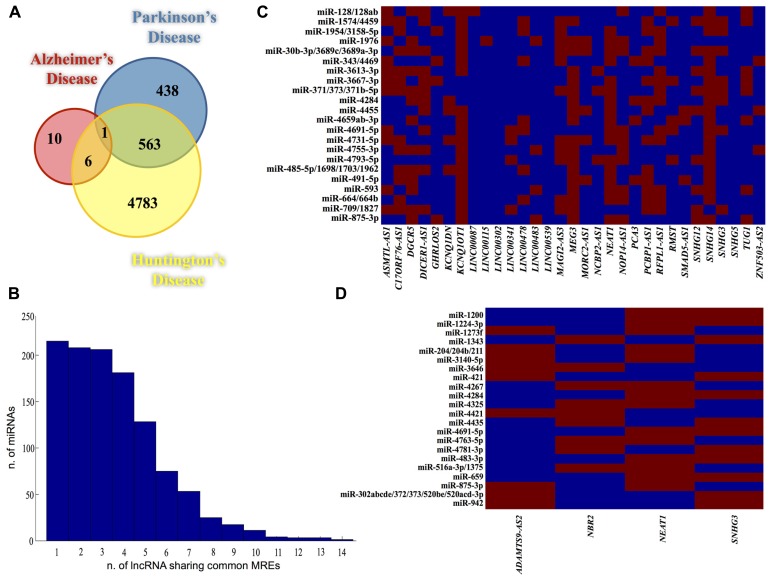
**(A)** Venn diagram showing intersections of DE genes in all three NDs. Colored circles contain the number of genes *per* disease. **(B)** Bar graph showing the distribution of all the miRNAs binding sites (*y-*axis) identified in lncRNAs DE in HD (*x*-axis) which bind the same miRNA. Matrixes built with the lncRNAs and the restricted set of miRNAs (having common binding sites within lncRNAs sequences), for HD **(C)** and PD **(D)**. Red and blue squares indicate the presence and the absence (respectively) of miRNA binding within the target lncRNA.

Therefore, we first identified the subset of genes differentially expressed in AD, PD, and HD, retrieving datasets from Gene Expression Atlas database^2^ (accession n. E-MTAB-62, E-GEOD-3790, E-GEOD-1751, E-AFMX-6, E-GEOD-7621, E-GEOD-7307, E-GEOD-20295, E-GEOD-20168, E-MEXP-2280, E-GEOD-6613). Particularly, only genes with a statistical significance of differential expression inferred from at least two independent experiments were used. As shown in **Figure 1A**, these datasets consisted of 17, 1002, and 5361 genes, for AD, PD, and HD, respectively. Interestingly, by using a bootstrap resampling procedure (10^5^ iterations), a significant overlap (563 genes; *p* ≪ 0.01) was disclosed between genes DE in PD and HD (**Figure 1A**), showing they may represent crucial genes in the etiology of neurodegeneration. Moreover, in line with the notion that common genes with proven involvement in cancer and in NDs are deregulated in both conditions (Morris et al., 2010; Plun-Favreau et al., 2010; Du and Pertsemlidis, 2011), pathway analysis (performed using PANTHER; Thomas et al., 2003) revealed a significant overlap with cancer hallmarks, including apoptosis, p53, Ras, PDGF, FGF, EGF, and MAPK signaling pathways (data not shown).

Since pseudogenes have been shown to act as miRNAs sponges in cancer we evaluated such finding also in NDs. Thus, we intersected the above-described datasets of DE genes in NDs with a full list of human pseudogenes retrieved from HUGO Gene Nomenclature Committee (HGNC) database. The intersection revealed that 49, 1, and 10 pseudogenes are DE in HD, AD, and PD, respectively. Thus, 3′ UTR sequences of pseudogenes and their parent genes were downloaded from University of California Santa Cruz (UCSC) and aligned by using BLAT algorithm to assess sequence identity. Only pseudogenes’ sequences showing high homology (95–99%) were used as described below. The 3′ UTRs of some parent genes aligned outside the boundaries of their annotated cognate pseudogenes, indicated the need to revise annotations. In such cases, we used for further computational analysis the matching genomic sequences. Therefore, FASTA sequences of selected pseudogenes and the 3′ UTRs of parent genes were independently scanned for the presence of miRNA binding sites using a TargetScan perl script (Lewis et al., 2005). Pseudogenes with only one miRNA binding site were excluded from further analyses. Analyzed pseudogene/gene pairs – in each ND – are listed in **Table 1**.

**Table 1:** Pseudogenes deregulated in HD, PD and AD sharing common miRNA binding sites with their parent genes.

Pseudogene/gene pair	miRNA family		
	Pseudogene	Gene	*n* common (%)
Huntington’s disease			
BCRP2/BCR*	41	71	38 (92.7)
BZW1P2/BZW1*	4	50	4 (100.0)
*CES1P1/CES1*	6	6	6 (100.0)
*CHCHD2P2/CHCHD2*	4	5	2 (50.0)
COX7A2P2/COX7A2*	3	5	2 (66.7)
*DGKZP1/DGKZ*	14	16	14 (100.0)
EEF1A1P5/EEF1A1*	9	64	8 (88.9)
*EIF2S2P4/EIF2S2*	6	33	6 (100.0)
*ETF1P1/ETF1*	24	59	19 (79.2)
*FABP5P1/FABP5*	3	2	2 (66.7)
*FAM108A3P/FAM108A1*	6	7	6 (100.0)
FAM115B/FAM115A*	76	76	74 (97.4)
GBP1P1/GBP1*	39	33	24 (61.5)
HIGD1AP14/HIGD1A*	19	56	14 (73.7)
HMGA1P4/HMGA1*	2	36	1 (50.0)
HMGB1P1/HMGA1*	42	36	29 (69.0)
*HMGB1P10/HMGB1*	15	48	13 (86.7)
*HMGB1P5/HMGB1*	14	48	13 (92.9)
*HMGN1P36/HMGN1*	11	11	10 (90.9)
*HMGN2P3/HMGN2*	12	24	12 (100.0)
*HNRNPA3P1/HNRNPA3*	53	118	5 (9.4)
*HSD17B7P2/HSD17B7*	7	9	6 (85.7)
*HTR7P1/HTR7*	35	43	17 (48.6)
MT1P2/MT1G*	2	4	2 (100.0)
MT1P2/MT1H*	2	5	2 (100.0)
*NANOGP8/NANOG*	18	17	17 (94.4)
*NSUN5P1/NSUN5*	15	39	12 (80.0)
*PI4KAP2/PI4KA*	7	7	5 (71.4)
*POU5F1P4/POU5F1*	7	9	6 (85.7)
PPP1R2P3/PPP1R2*	58	55	38 (65.5)
PTENP1/PTEN*	23	91	21 (91.3)
*RBBP4P4/RBBP4*	13	147	11 (84.6)
RBMS1P1/RBMS1*	15	52	15 (100.0)
RHOQP2/RHOQ*	52	93	48 (92.3)
*RP9P/RP9*	16	19	15 (93.8)
RPLP0P6/RPLP0*	2	2	1 (50.0)
S100A11P1/S100A11*	6	5	3 (50.0)
SKP1P1/SKP1*	25	35	18 (72.0)
*TLK2P1/TLK2*	20	64	19 (95.0)
VDAC1P1/VDAC1*	21	26	20 (95.2)
VEZF1P1/VEZF1*	64	85	53 (82.8)
YWHAZP3/YWHAZ*	11	54	8 (72.7)
*ZFAND6P1/ZFAND6*	15	16	14 (93.3)
**Parkinson’s disease**			
*CHCHD2P2/CHCHD2*	4	5	2 (50.0)
*HSD17B7P2/HSD17B7*	7	9	6 (85.7)
*NSUN5P1/NSUN5*	15	39	12 (80.0)
*NSUN5P2/NSUN5*	13	39	11 (84.6)
*PHC1P1/PHC1*	47	46	46 (87.9)
*PPP1R2P3/PPP1R2*	58	55	38 (65.5)
*RBMXP2/RBMX*	26	59	22 (84.6)
**Alzheimer’s disease**		
*CRYBB2P1/CRYBB2*	6	5	5 (83.3)

* Genes DE in the related disease.

This analysis revealed that pseudogenes deregulated in HD, AD, and PD, share (on average) about 80% of miRNA binding sites with their parent genes, suggesting these highly expressed – but untranslated – transcripts may represent novel ceRNAs, possibly subtracting a relevant fraction of common miRNAs to the physiological regulation of their parent genes. Interestingly, our analysis revealed that two pseudogenes, *PTENP1* and *POU5F1P4* – recently described as ceRNAs in cancer (Poliseno et al., 2010) – are differentially expressed in NDs and share a very significant fraction of MREs (about 90%) with their parent genes (see **Table 1**).

Our findings strengthen the hypothesis of a novel convergent ceRNA-mediated regulatory mechanism, underlying both cancerogenesis and neurodegenerative process. We cannot exclude that over-expression of such pseudogenes may subtract a pool of miRNAs not only to their parent genes, but they may also contribute to a more global gene deregulation, accounting for disease etiology. Systematic analysis of DE pseudogenes in NDs, and further targeted functional studies are needed to confirm these observations.

### LncRNA IN NEURODEGENERATIVE DISEASES

Long non-coding RNAs are a numerous class of non-protein coding transcripts longer than 200 nucleotides. Prior studies and, more recently, transcriptomic analyses by Next Generation Sequencing (NGS), indicate the lncRNAs are as abundant as mRNAs (Carninci et al., 2005; Guttman et al., 2009; Cabili et al., 2011). Given their proven key role in many biological processes and their restricted expression pattern in specific brain regions (Mercer et al., 2008), it is reasonable to speculate they may be altered in NDs and be involved in their etiology (Johnson, 2012; Niland et al., 2012). Furthermore, the hypothesis that lncRNAs could sequester miRNAs and act as ceRNAs (Cesana et al., 2011; Salmena et al., 2011), suggests a novel fascinating role for them in NDs.

In light of these considerations, we examined lncRNAs differentially expressed in NDs, similarly to pseudogenes analysis. By using an *in silico* approach and a list of human lncRNAs obtained from HGNC database, we identified 31, 4, and 1 lncRNAs DE in HD, PD, and AD, respectively. Since they are alternatively spliced, we retrieved the sequences corresponding to all splicing transcripts (222, 5, and 1 Ensembl transcripts for HD, PD, and AD, respectively) and we scanned them for the presence of miRNA binding sites. In AD, the only lncRNA significantly DE was *BACE1-AS *whose role in Alzheimer’s pathogenesis has been already reported (Faghihi et al., 2008, 2010). Our analysis revealed *BACE1-AS *has predicted binding sites for 18 miRNAs, some of with proven association to AD, such as let-7, mir-127, mir-93a (Chan and Kocerha, 2012; Lehmann et al., 2012), suggesting it may be an intriguing ceRNA candidate in AD etiology. The distribution of MREs within lncRNAs DE in HD (**Figure 1B**) and PD was evaluated in order to identify the lncRNAs sharing a pool of common MREs. By using a bootstrap resampling procedure we created random sets of lncRNAs that underwent the same miRNA analysis. We measured their MREs distributions observing they significantly differ from our observations (*p* < 0.05).

Moreover, given the large number of lncRNAs DE in HD and PD, we used random datasets also to set two thresholds on the number of lncRNAs sharing common MREs, whose values were 10 for HD and 2 for PD. Such thresholds were used to select – for further analysis – a restricted pool of miRNAs whose seeds match at least 10 and 2 lncRNAs analyzed for HD and PD, respectively. Thus, we built two matrixes (one for disease) with all the lncRNAs altered in a specific disease and its related stringent set of miRNAs (**Figures 1C and 1D** for HD and PD, respectively).

Although predictive and computationally-based, our analysis shows that lncRNAs deregulated in NDs share a pool of MREs, suggesting such long untranslated transcripts may represent a previously undetected source of competitive binding sites also for brain-specific miRNAs, thus potentially acting as ceRNAs.

## PERSPECTIVES AND CONCLUSIONS

Growing interest in understanding the basis of neurodegenerative processes has led to significant steps forward, even though many underlying molecular aspects are still unknown. The identification of disease-causing mutations in Mendelian forms of NDs and genome-wide associations studies have only partially provided satisfactory explanation to disease pathogenesis, whereas gene expression studies, and the analysis of their regulation, are currently giving a significant contribution to better understand NDs. Particularly, miRNAs and other ncRNAs are showing relevant roles in neural cell plasticity as well as in neurodegenerative processes (Junn and Mouradian, 2012).

In cancer, recent evidences show that untranslated transcripts, pseudogenes, and presumably lncRNAs, named ceRNAs, compete for a pool of miRNAs acting as endogenous sponges and regulating parent genes and other mRNAs (Poliseno et al., 2010; Karreth et al., 2011; Salmena et al., 2011; Tay et al., 2011). Such findings are likely to have broader implications for other diseases and cellular processes, largely beyond the regulation of few genes in cancer.

Therefore, given that NDs and cancer share common causative genes and altered signaling molecular pathways, even considering the crucial role of miRNAs in neurogenesis- and cancerogenesis-related processes, we have proposed and computationally predicted both pseudogenes and lncRNAs may be involved in the etiology of AD, HD, and PD, acting as ceRNAs.

In such NDs, independent analysis of DE lncRNAs, pseudogenes, and parent genes, revealed they contain a huge number of shared MREs, potentially representing miRNAs sponges. It suggests that ceRNAs may represent the rule, rather than the exception, also in the etiology of NDs. Our observations indicate that a ceRNA-based regulatory mechanism might be shared between neurodegenerative and cancerous processes, and we cannot exclude that similar complex regulatory networks may also underlie other human complex diseases. However, studying pseudogenes is challenging due to the high sequence identity with their parent genes, and genome-wide expression studies may report conflicting results about pseudogenes expression. The introduction of NGS, particularly of RNA sequencing, is substantially contributing to overcome some technological challenges for the transcriptome analysis (Cloonan et al., 2008; Mortazavi et al., 2008; Costa et al., 2010, 2011) also for studying expressed pseudogenes. We believe this technology will increasingly help researchers to encrypt the novel ceRNAs code, giving an incredible boost to understand this new “language.”

Finally, targeted functional studies are clearly needed to validate and confirm this predictive study, even though we believe that ceRNAs have traced a novel revolutionary route in the landscape of human genetics.

## Conflict of Interest Statement

The authors declare that the research was conducted in the absence of any commercial or financial relationships that could be construed as a potential conflict of interest.

## Footnotes

1 http://www.mir2disease.org

2 http://www.ebi.ac.uk/gxa/

## References

[B1] Bartel D. P. (2009). MicroRNAs: target recognition and regulatory functions. *Cell* 136 215–233 19167326 10.1016/j.cell.2009.01.002 PMC3794896

[B2] Cabili M. N. Trapnell C. Goff L. Koziol M. Tazon-Vega B. Regev A. et al. (2011). Integrative annotation of human large intergenic noncoding RNAs reveals global properties and specific subclasses. *Genes Dev.* 25 1915–1927 21890647 10.1101/gad.17446611 PMC3185964

[B3] Carninci P. Kasukawa T. Katayama S. Gough J. Frith M. C. Maeda N. (2005). The transcriptional landscape of the mammalian genome. *Science* 309 1559–15563 16141072 10.1126/science.1112014

[B4] Cesana M. Cacchiarelli D. Legnini I. Santini T. Sthandier O. Chinappi M. et al. (2011). A long noncoding RNA controls muscle differentiation by functioning as a competing endogenous RNA. *Cell* 147 358–369 22000014 10.1016/j.cell.2011.09.028 PMC3234495

[B5] Chan A. W. Kocerha J. (2012). The path to microRNA therapeutics in psychiatric and neurodegenerative disorders. *Front. Genet. *3:82. 10.3389/fgene.2012.00082 PMC3354561 22629284

[B6] Chartier-Harlin M. C. Kachergus J. Roumier C. Mouroux V. Douay X. Lincoln S. et al. (2004). Alpha-synuclein locus duplication as a cause of familial Parkinson’s disease. *Lancet* 364 1167–1169 15451224 10.1016/S0140-6736(04)17103-1

[B7] Chi S. W. Zang J. B. Mele A. Darnell R. B. (2009). Argonaute HITS-CLIP decodes microRNA-mRNA interaction maps. *Nature* 460 479–486 19536157 10.1038/nature08170 PMC2733940

[B8] Cloonan N. Forrest A. R. Kolle G. Gardiner B. B. Faulkner G. J. Brown M. K. (2008). Stem cell transcriptome profiling via massive-scale mRNA sequencing. *Nat. Methods* 5 613–619 18516046 10.1038/nmeth.1223

[B9] Cogswell J. P. Ward J. Taylor I. A. Waters M. Shi Y. Cannon B. et al. (2008). Identification of miRNA changes in Alzheimer’s disease brain and CSF yields putative biomarkers and insights into disease pathways. *J. Alzheimers Dis.* 14 27–41 18525125 10.3233/jad-2008-14103

[B10] Costa V. Angelini C. D’Apice L. Mutarelli M. Casamassimi A. Sommese L. et al. (2011). Massive-scale RNA-Seq analysis of non ribosomal transcriptome in human trisomy 21. *PLoS ONE* 6 e18493 10.1371/journal.pone.0018493 PMC3080369 21533138

[B11] Costa V. Angelini C. De Feis I. Ciccodicola A. (2010). Uncovering the complexity of transcriptomes with RNA-Seq. *J. Biomed. Biotechnol.* 2010 853916 10.1155/2010/853916 PMC2896904 20625424

[B12] Costa V. Aprile M. Esposito R. Ciccodicola A. (2012). RNA-Seq and human complex diseases: recent accomplishments and future perspectives. *Eur. J. Hum. Genet.* 10.1038/ejhg.2012.129 [Epub ahead of print] PMC3548270 22739340

[B13] Du L. Pertsemlidis A. (2011). Cancer and neurodegenerative disorders: pathogenic convergence through microRNA regulation. *J. Mol. Cell Biol.* 3 176–180 21278200 10.1093/jmcb/mjq058 PMC3104012

[B14] Ertekin-Taner N. (2011). Gene expression endophenotypes: a novel approach for gene discovery in Alzheimer’s disease. *Mol. Neurodegener.* 6 31 10.1186/1750-1326-6-31 PMC3113300 21569597

[B15] Esteller M. (2011). Non-coding RNAs in human disease. *Nat. Rev. Genet.* 12 861–874 22094949 10.1038/nrg3074

[B16] Faghihi M. A. Modarresi F. Khalil A. M. Wood D. E. Sahagan B. G. Morgan T. E. et al. (2008). Expression of a noncoding RNA is elevated in Alzheimer’s disease and drives rapid feed-forward regulation of beta-secretase. *Nat. Med.* 14 723–730 18587408 10.1038/nm1784 PMC2826895

[B17] Faghihi M. A. Zhang M. Huang J. Modarresi F. Van der Brug M. P. Nalls M. A. (2010). Evidence for natural antisense transcript-mediated inhibition of microRNA function. *Genome Biol.* 11 R56 10.1186/gb-2010-11-5-r56 PMC2898074 20507594

[B18] Geekiyanage H. Jicha G. A. Nelson P. T. Chan C. (2012). Blood serum miRNA: non-invasive biomarkers for Alzheimer’s disease. *Exp. Neurol.* 235 491–496 22155483 10.1016/j.expneurol.2011.11.026 PMC3361462

[B19] Ghose J. Sinha M. Das E. Jana N. R. Bhattacharyya N. P. (2011). Regulation of miR-146a by RelA/NFkB and p53 in STHdh(Q111)/Hdh(Q111) cells, a cell model of Huntington’s disease. *PLoS ONE* 6 e23837 10.1371/journal.pone.0023837 PMC3162608 21887328

[B20] Guo H. Ingolia N. T. Weissman J. S. Bartel D. P. (2010). Mammalian microRNAs predominantly act to decrease target mRNA levels. *Nature* 466 835–840 20703300 10.1038/nature09267 PMC2990499

[B21] Guttman M. Amit I. Garber M. French C. Lin M. F. Feldser D. et al. (2009). Chromatin signature reveals over a thousand highly conserved large non-coding RNAs in mammals. *Nature* 458 223–227 19182780 10.1038/nature07672 PMC2754849

[B22] Hébert S. S. Horré K. Nicolaї L. Bergmans B. Papadopoulou A. S. Delacourte A. et al. (2009). MicroRNA regulation of Alzheimer’s Amyloid precursor protein expression. *Neurobiol. Dis.* 33 422–428 19110058 10.1016/j.nbd.2008.11.009

[B23] Johnson R. (2012). Long non-coding RNAs in Huntington’s disease neurodegeneration. *Neurobiol. Dis.* 46 245–254 22202438 10.1016/j.nbd.2011.12.006

[B24] Junn E. Mouradian M. M. (2012). MicroRNAs in neurodegenerative diseases and their therapeutic potential. *Pharmacol. Ther.* 133 142–150 22008259 10.1016/j.pharmthera.2011.10.002 PMC3268953

[B25] Kapsimali M. Kloosterman W. P. de Bruijn E. Rosa F. Plasterk R. H. Wilson S. W. (2007). MicroRNAs show a wide diversity of expression profiles in the developing and mature central nervous system. *Genome Biol.* 8 R173 10.1186/gb-2007-8-8-r173 PMC2375003 17711588

[B26] Karreth F. A. Tay Y. Perna D. Ala U. Tan S. M. Rust A. G. et al. (2011). In vivo identification of tumor-suppressive PTEN ceRNAs in an oncogenic BRAF-induced mouse model of melanoma. *Cell* 147 382–395 22000016 10.1016/j.cell.2011.09.032 PMC3236086

[B27] Kim J. Inoue K. Ishii J. Vanti W. B. Voronov S. V. Murchison E. et al. (2007). A MicroRNA feedback circuit in midbrain dopamine neurons. *Science* 317 1220–1224 17761882 10.1126/science.1140481 PMC2782470

[B28] Lehmann S. M. Krüger C. Park B. Derkow K. Rosenberger K. Baumgart J. et al. (2012). An unconventional role for miRNA: let-7 activates Toll-like receptor 7 and causes neurodegeneration. *Nat. Neurosci.* 15 827–835 22610069 10.1038/nn.3113

[B29] Lewis B. P. Burge C. B. Bartel D. P. (2005). Conserved seed pairing, often flanked by adenosines, indicates that thousands of human genes are microRNA targets. *Cell* 120 15–20 15652477 10.1016/j.cell.2004.12.035

[B30] Licatalosi D. D. Mele A. Fak J. J. Ule J. Kayikci M. Chi S. W. et al. (2008). HITS-CLIP yields genome-wide insights into brain alternative RNA processing. *Nature* 456 464–469 18978773 10.1038/nature07488 PMC2597294

[B31] Liu W. Liu C. Zhu J. Shu P. Yin B. Gong Y. et al. (2012). MicroRNA-16 targets amyloid precursor protein to potentially modulate Alzheimer’s associated pathogenesis in SAMP8 mice. *Neurobiol. Aging* 33 522–534 20619502 10.1016/j.neurobiolaging.2010.04.034

[B32] Long J. M. Lahiri D. K. (2011). Current drug targets for modulating Alzheimer’s amyloid precursor protein: role of specific micro-RNA species. *Curr. Med. Chem.* 18 3314–3321 21728971 10.2174/092986711796504592

[B33] Lukiw W. J. (2007). Micro-RNA speciation in fetal, adult and Alzheimer’s disease hippocampus. *Neuroreport* 18 297–300 17314675 10.1097/WNR.0b013e3280148e8b

[B34] Margis R. Margis R. Rieder C. R. (2011). Identification of blood microRNAs associated to Parkinsonis disease. *J. Biotechnol.* 152 96–101 21295623 10.1016/j.jbiotec.2011.01.023

[B35] Martí E. Pantano L. Bañez-Coronel M. Llorens F. Miñones-Moyano E. Porta S. et al. (2010). A myriad of miRNA variants in control and Huntington’s disease brain regions detected by massively parallel sequencing. *Nucleic Acids Res.* 38 7219–7235 20591823 10.1093/nar/gkq575 PMC2978354

[B36] Mercer T. R. Dinger M. E. Sunkin S. M. Mehler M. F. Mattick J. S. (2008). Specific expression of long noncoding RNAs in the mouse brain. *Proc. Natl. Acad. Sci. U.S.A.* 105 716–721 18184812 10.1073/pnas.0706729105 PMC2206602

[B37] Miñones-Moyano E. Porta S. Escaramís G. Rabionet R. Iraola S. Kagerbauer B. et al. (2011). MicroRNA profiling of Parkinson’s disease brains identifies early downregulation of miR-34b/c which modulate mitochondrial function. *Hum. Mol. Genet.* 20 3067–3078 21558425 10.1093/hmg/ddr210

[B38] Miska E. A. Alvarez-Saavedra E. Townsend M. Yoshii A. Sestan N. Rakic P. (2004). Microarray analysis of microRNA expression in the developing mammalian brain. *Genome Biol.* 5 R68 10.1186/gb-2004-5-9-r68 PMC522875 15345052

[B39] Morris L. G. Veeriah S. Chan T. A. (2010). Genetic determinants at the interface of cancer and neurodegenerative disease. *Oncogene* 29 3453–3464 20418918 10.1038/onc.2010.127 PMC3005561

[B40] Mortazavi A. Williams B. A. McCue K. Schaeffer L. Wold B. (2008). Mapping and quantifying mammalian transcriptomes by RNA-Seq. *Nat. Methods* 5 621–628 18516045 10.1038/nmeth.1226 PMC13303166

[B41] Niland C. N. Merry C. R. Khalil A. M. (2012). Emerging roles for long non-coding RNAs in cancer and neurological disorders. *Front. Genet.* 3 25 10.3389/fgene.2012.00025 PMC3286759 22375145

[B42] Noble E. P. (2000). Addiction and its reward process through polymorphisms of the D2 dopamine receptor gene: a review. *Eur. Psychiatry* 15 79–89 10881203 10.1016/s0924-9338(00)00208-x

[B43] Packer A. N. Xing Y. Harper S. Q. Jones L. Davidson B. L. (2008). The bifunctional microRNA miR-9/miR-9* regulates REST and CoREST and is downregulated in Huntington’s disease. *J. Neurosci.* 28 14341–14346 19118166 10.1523/JNEUROSCI.2390-08.2008 PMC3124002

[B44] Patel N. Hoang D. Miller N. Ansaloni S. Huang Q. Rogers J. T. et al. (2008). MicroRNAs can regulate human APP levels. *Mol. Neurodegener.* 3 10 10.1186/1750-1326-3-10 PMC2529281 18684319

[B45] Plun-Favreau H. Lewis P. A. Hardy J. Martins L. M. Wood N. W. (2010). Cancer and neurodegeneration: between the devil and the deep blue sea. *PLoS Genet.* 6 e1001257 10.1371/journal.pgen.1001257 PMC3009676 21203498

[B46] Podlisny M. B. Lee G. Selkoe D. J. (1987). Gene dosage of the amyloid beta precursor protein in Alzheimer’s disease. *Science* 238 669–671 2960019 10.1126/science.2960019

[B47] Poliseno L. Salmena L. Zhang J. Carver B. Haveman W. J. Pandolfi P. P. (2010). A coding-independent function of gene and pseudogene mRNAs regulates tumour biology. *Nature* 465 1033–1038 20577206 10.1038/nature09144 PMC3206313

[B48] Qureshi I. A. Mehler M. F. (2012). Emerging roles of non-coding RNAs in brain evolution, development, plasticity and disease. *Nat. Rev. Neurosci.* 13 528–541 22814587 10.1038/nrn3234 PMC3478095

[B49] Rajewsky N. (2006). microRNA target predictions in animals. *Nat. Genet.* 38(Suppl) S8–S13 16736023 10.1038/ng1798

[B50] Rajewsky N. Socci N. D. (2004). Computational identification of microRNA targets. *Dev. Biol.* 267 529–535 15013811 10.1016/j.ydbio.2003.12.003

[B51] Rovelet-Lecrux A. Hannequin D. Raux G. Le Meur N. Laquerriére A. Vital A. et al. (2006). APP locus duplication causes autosomal dominant early-onset Alzheimer disease with cerebral amyloid angiopathy. *Nat. Genet.* 38 24–26 16369530 10.1038/ng1718

[B52] Salmena L. Poliseno L. Tay Y. Kats L. Pandolfi P. P. (2011). A ceRNA hypothesis: the Rosetta Stone of a hidden RNA language? *Cell* 146 353–368 21802130 10.1016/j.cell.2011.07.014 PMC3235919

[B53] Schofield C. M. Hsu R. Barker A. J. Gertz C. C. Blelloch R. Ullian E. M. (2011). Monoallelic deletion of the microRNA biogenesis gene Dgcr8 produces deficits in the development of excitatory synaptic transmission in the prefrontal cortex. *Neural Dev.* 6 11 10.1186/1749-8104-6-11 PMC3082233 21466685

[B54] Seitz H. (2009). Redefining microRNA targets. *Curr. Biol.* 19 870–873 19375315 10.1016/j.cub.2009.03.059

[B55] Singleton A. B. Farrer M. Johnson J. Singleton A. Hague S. Kachergus J. et al. (2003). alpha-Synuclein locus triplication causes Parkinson’s disease. *Science* 302 841 10.1126/science.1090278 14593171

[B56] Sutherland G. T. Janitz M. Kril J. J. (2011). Understanding the pathogenesis of Alzheimer’s disease: will RNA-Seq realize the promise of transcriptomics? *J. Neurochem.* 116 937–946 21175619 10.1111/j.1471-4159.2010.07157.x

[B57] Tay Y. Kats L. Salmena L. Weiss D. Tan S. M. Ala U. et al. (2011). Coding-independent regulation of the tumor suppressor PTEN by competing endogenous mRNAs. *Cell* 147 344–357 22000013 10.1016/j.cell.2011.09.029 PMC3235920

[B58] Thomas P. D. Kejariwal A. Campbell M. J. Mi H. Diemer K. Guo N. et al. (2003). PANTHER: a browsable database of gene products organized by biological function, using curated protein family and subfamily classification. *Nucleic Acids Res.* 31 334–341 12520017 10.1093/nar/gkg115 PMC165562

